# Inhibition of NLRP3 Inflammasome Activation by 3H-1,2-Dithiole-3-Thione: A Potential Therapeutic Approach for Psoriasis Treatment

**DOI:** 10.3390/ijms241713528

**Published:** 2023-08-31

**Authors:** Meng-Chieh Shih, Chia-Ling Li, En-Chih Liao, Chung-Yang Yen, Ling-Jung Yen, Kai-Chun Wang, Ling-Ying Lu, Ting-Yu Chou, Ying-Chin Chen, Sheng-Jie Yu

**Affiliations:** 1 Division of Allergy, Immunology, and Rheumatology, Department of Internal Medicine, Kaohsiung Veterans General Hospital, Kaohsiung 813, Taiwan; mcshih@vghks.gov.tw (M.-C.S.); ljung@vghks.gov.tw (L.-J.Y.); kcwang@vghks.gov.tw (K.-C.W.); lylu@vghks.gov.tw (L.-Y.L.); ycchen5741596@vghks.gov.tw (Y.-C.C.); 2 Children’s Medical Center, Taichung Veterans General Hospital, Taichung 407, Taiwan; lingboxer@gmail.com; 3 Institute of Biomedical Sciences, MacKay Medical College, New Taipei City 252, Taiwan; enchih@mmc.edu.tw; 4 Department of Medicine, MacKay Medical College, New Taipei City 252, Taiwan; 5 Department of Dermatology, Taichung Veterans General Hospital, Taichung 407, Taiwan; vernayen@yahoo.com.tw; 6 School of Medicine, National Yang Ming Chiao Tung University, Taipei 112, Taiwan; 7 Integrated Care Center of Psoriatic Disease, Taichung Veterans General Hospital, Taichung 407, Taiwan; 8 The Doctoral Program of Clinical and Experimental Medicine, National Sun Yat-Sen University, Kaohsiung 804, Taiwan; 9 Department of Medical Research, Taichung Veterans General Hospital, Taichung 407, Taiwan; b0980468524@gmail.com; 10 Institute of Biomedical Sciences, National Sun Yat-Sen University, Kaohsiung 804, Taiwan; 11 Institute of Biomedical Sciences, College of Life Sciences, National Chung Hsing University, Taichung 407, Taiwan

**Keywords:** psoriasis, 3H-1,2-dithiole-3-thione (D3T), NLRP3 inflammasome, keratinocytes, inflammation

## Abstract

Psoriasis is a chronic autoimmune skin disease with a significant impact on quality of life and potential for severe comorbidities. Inflammation in the skin is induced by immune cells that overexpress pro-inflammatory cytokines, with the Th17 cell playing a crucial role. NLRP3 inflammasome activation is associated with inflammatory diseases and abnormal T cell differentiation. 3H-1,2-dithiole-3-thione (D3T), isolated from cruciferous vegetables, has anti-inflammatory effects and inhibits Th17 differentiation. This study aimed to investigate how D3T reduces skin inflammation and modulates Th17 cell differentiation by inhibiting NLRP3 inflammasome activation. In an imiquimod-induced psoriasis mouse model, D3T treatment demonstrated significant reductions in ear thickness, skin redness, and scaling compared to a control group. Our study also observed decreased expression of ki-67, NLRP3 inflammasome, and cleaved caspase-1 in skin samples, reduced levels of IL-6 and IL-17A in serum samples, and inhibition of Th17 differentiation after D3T application. D3T could also inhibit the expression of NLRP3, caspase-1, and IL-1β in TNF-α stimulated HaCaT cells. The mechanical study also revealed that D3T could inhibit NLRP3 inflammasome activation by inhibiting the JNK pathway in HaCaT cells. These results indicate that targeting NLRP3 inflammasome activation is a promising strategy in the treatment of psoriasis.

## 1. Introduction

Psoriasis is a chronic inflammatory skin disease characterized by erythematous plaques with overlying coarse scale and a variety of clinical manifestations. Both males and females can be diagnosed with psoriasis, with the estimated prevalence ranging from 0.5% to 11.4% in adults [1]. Psoriasis significantly diminishes one’s quality of life, leading to a loss of confidence, negative outlook, reduced social interaction, and the potential development of psychological disorders among patients. The exact cause of psoriasis is not fully understood, though it has been reported that proinflammatory cytokines such as IL-17A, TNF-α, and IFN-γ play an important role in the pathogenesis of psoriasis [2]. The immune system of psoriasis patients loses its tolerance, causing the immune cells to attack healthy epidermal cells, thus accelerating the rate of over production of unwell differentiated skin cells and the accumulation of dead skin cells. Inflammasomes have been identified one after another during the past decade and consist of multiple protein complex in cytoplasm [3]. The inflammasomes are able to respond to stimuli such as pathogens and proinflammatory cytokines [4]. Among these inflammasomes, the NLRP3 inflammasome is the most studied, and is correlated to many inflammatory diseases such as psoriasis [5]. The pathogenic T cells which secrete cytokines can activate the NLRP3 inflammasome of keratinocyte, then caspase-1 mediated pro IL-1β cleavage will produce active IL-1β exacerbating skin inflammation [6]. The current treatment of psoriasis mostly focuses on immune cell modulation; however, targeting the inhibition of skin inflammation is also important. Thus, further investigation on regulating NLRP3 inflammasome of keratinocyte activation and developing a novel strategy for fighting psoriasis are needed. 3H-1,2-dithiole-3-thione (D3T) is one of the sulfur-containing dithiolethiones which can be found in cruciferous vegetables and has been previously reported to activate the Nrf2 signaling pathway and help to decrease intracellular oxidative stress [7,8]. D3T has been used to treat autoimmune disease and experimental autoimmune encephalomyelitis (EAE), and can inhibit pathologic Th17 cells differentiation to downregulate disease scores in EAE animal models [9]. It has been reported that regulating oxidative stress could inhibit inflammasome activation [10]; however, the effect of D3T on inhibiting inflammasome activation remains unknown and little is known about the correlation between inflammasome activation and psoriasis. In this study, we aimed to investigate the effects of D3T in the treatment of psoriasis in imiquimod (IMQ)-induced psoriasis animal models, as well as the effect which D3T has on reducing proinflammatory cytokine expression through inhibiting NLRP3 inflammasome activation.

## 2. Results

### 2.1. D3T Treatment Alleviated IMQ-Induced Psoriasis-like Skin Inflammation in BALB/c Mice

**Figure 1:**
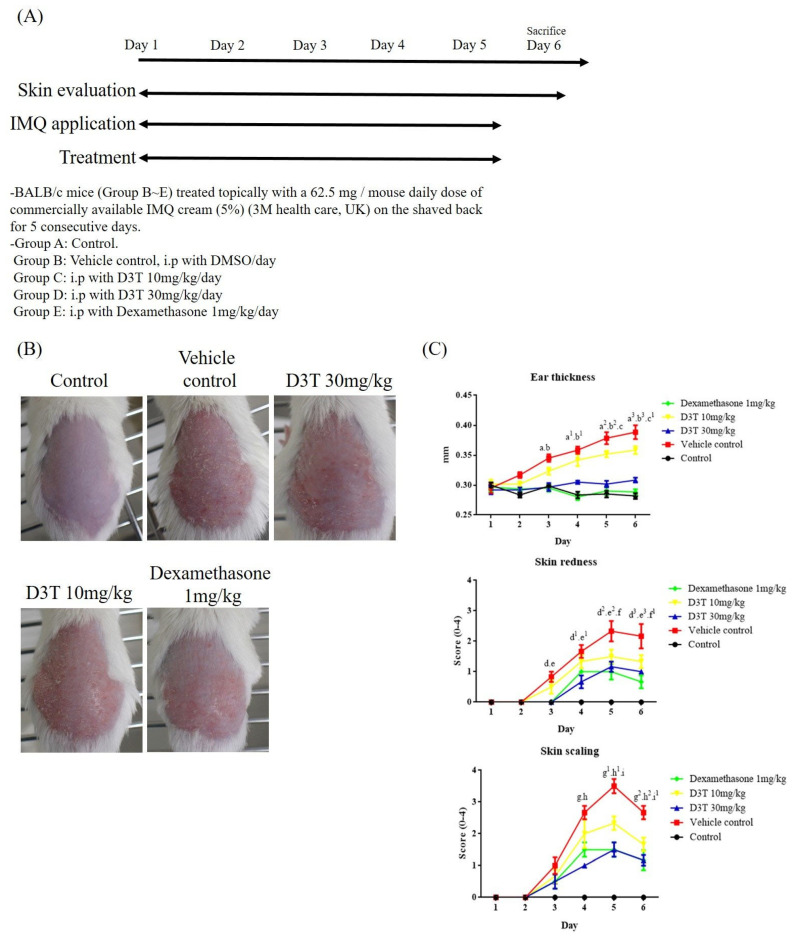
D3T ameliorated psoriasis in an IMQ-induced psoriasis mouse model. Except for the control group, all mice received a topical application of IMQ on their shaved backs for five consecutive days. In the treatment group, mice were treated with a vehicle control (DMSO), D3T (10, 30 mg/kg), and dexamethasone (1 mg/kg) on the same day that topical IMQ was applied. Mice were sacrificed on Day 6. (**A**) The experiment protocols. (**B**) Psoriasis lesions of the dorsal skin were present on Day 6. (**C**) Skin redness and scaling were evaluated each day, with ear thickness being measured using an electronic gauge. Data are expressed as the mean ± SEM (n = 6 in each group). a—D3T 30 mg/kg compared to vehicle control on day 3, *p* < 0.0001; a^1^—D3T 30 mg/kg compared to vehicle control on day 4, *p* < 0.0001; a^2^—D3T 30 mg/kg compared to vehicle control on day 5, *p* < 0.0001; a^3^—D3T 30 mg/kg compared to vehicle control on day 6, *p* < 0.0001; b—Dexamethasone compared to vehicle control on day 3, *p* < 0.0001; b^1^—Dexamethasone compared to vehicle control on day 4, *p* < 0.0001; b^2^—Dexamethasone compared to vehicle control on day 5, *p* < 0.0001; b^3^—Dexamethasone compared to vehicle control on day 6, *p* < 0.0001; c—D3T 10 mg/kg compared to vehicle control on day 5, *p* < 0.05; c^1^—D3T 10 mg/kg compared to vehicle control on day 5, *p* < 0.01; d—D3T 30 mg/kg compare to vehicle control on day 3, *p* < 0.01; d^1^—D3T 30 mg/kg compare to vehicle control on day 4, *p* < 0.0001; d^2^—D3T 30 mg/kg compare to vehicle control on day 5, *p* < 0.0001; d^3^—D3T 30 mg/kg compare to vehicle control on day 6, *p* < 0.0001; e—Dexamethasone compare to vehicle control on day 3, *p* < 0.01; e^1^—Dexamethasone compare to vehicle control on day 4, *p* < 0.05; e^2^—Dexamethasone compare to vehicle control on day 5, *p* < 0.0001; e^3^—Dexamethasone compare to vehicle control on day 6, *p* < 0.0001; f—D3T 10 mg/kg compare to vehicle control on day 5, *p* < 0.01; f^1^—D3T 10 mg/kg compare to vehicle control on day 5, *p* < 0.01; g—D3T 30 mg/kg compare to vehicle control on day 4, *p* < 0.0001; g^1^—D3T 30 mg/kg compare to vehicle control on day 5, *p* < 0.0001; g^2^—D3T 30 mg/kg compare to vehicle control on day 6, *p* < 0.0001; h—Dexamethasone compare to vehicle control on day 4, *p* < 0.0001; h^1^—Dexamethasone compare to vehicle control on day 5, *p* < 0.0001; h^2^—Dexamethasone compare to vehicle control on day 6, *p* < 0.0001; i—D3T 10 mg/kg compare to vehicle control on day 5, *p* < 0.0001; i^1^—D3T 10 mg/kg compare to vehicle control on day 5, *p* < 0.001.

**Figure 2:**
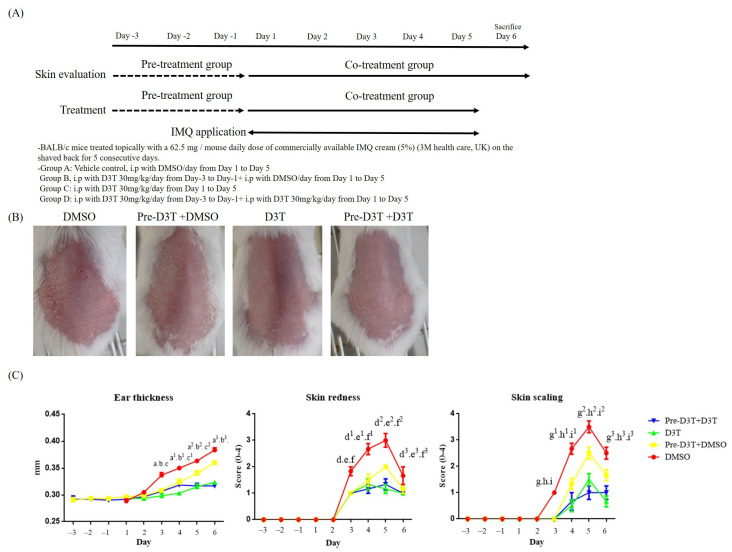
Pharmacodynamics of D3T on treating IMQ-induced psoriasis mouse model. All mice received a topical application of IMQ on their shaved backs for five consecutive days. In the treatment group, mice were treated with a vehicle control (DMSO), pretreated D3T (30 mg/kg) followed by DMSO treatment for 5 days, D3T (30 mg/kg), and pretreated D3T (30 mg/kg) followed by D3T (30 mg/kg) treatment for 5 days. Mice were sacrificed on Day 6. (**A**) The experiment protocols. (**B**) Psoriasis lesions of the dorsal skin were present on Day 6. (**C**) Skin redness and scaling were evaluated each day, with ear thickness being measured using an electronic gauge. Data are expressed as the mean ± SEM (n = 6 in each group). a—Pre-D3T + DMSO compared to DMSO on day 3, *p* < 0.0001, a^1^—Pre-D3T + DMSO compared to DMSO on day 4, *p* < 0.0001, a^2^—Pre-D3T + DMSO compared to DMSO on day 5, *p* < 0.0001, a^3^—Pre-D3T + DMSO compared to DMSO on day 6, *p* < 0.0001, b—D3T compared to DMSO on day 3, *p* < 0.0001, b^1^—D3T compared to DMSO on day 4, *p* < 0.0001, b^2^—D3T compared to DMSO on day 5, *p* < 0.0001, b^3^—D3T compared to DMSO on day 6, *p* < 0.0001, c—Pre-D3T + D3T compared to DMSO on day 3, *p* < 0.0001, c^1^—Pre-D3T + D3T compared to DMSO on day 4, *p* < 0.0001, c^2^—Pre-D3T + D3T compared to DMSO on day 5, *p* < 0.0001, c^3^—Pre-D3T + D3T compared to DMSO on day 6, *p* < 0.0001, d—Pre-D3T+DMSO compare to DMSO on day 3, *p* < 0.0001, d^1^—Pre-D3T+DMSO compare to DMSO on day 4, *p* < 0.0001, d^2^—Pre-D3T+DMSO compare to DMSO on day 5, *p* < 0.0001, d^3^—Pre-D3T+DMSO compare to DMSO on day 6, *p* < 0.05, e—D3T compare to DMSO on day 3, *p* < 0.0001, e^1^—D3T compare to DMSO on day 4, *p* < 0.0001, e^2^—D3T compare to DMSO on day 5, *p* < 0.0001, e^3^—D3T compare to DMSO on day 6, *p* < 0.001, f—Pre-D3T+D3T compare to DMSO on day 3, *p* < 0.0001, f^1^—Pre-D3T+D3T compare to DMSO on day 4, *p* < 0.0001, f^2^—Pre-D3T+D3T compare to DMSO on day 5, *p* < 0.0001, f^3^—Pre-D3T+D3T compare to DMSO on day 6, *p* < 0.001, g—Pre-D3T+DMSO compare to DMSO on day 3, *p* < 0.0001, g^1^—Pre-D3T+DMSO compare to DMSO on day 4, *p* < 0.0001, g^2^—Pre-D3T+DMSO compare to DMSO on day 5, *p* < 0.0001, g^3^—Pre-D3T+DMSO compare to DMSO on day 6, *p* < 0.001, h—D3T compare to DMSO on day 3, *p* < 0.0001, h^1^—D3T compare to DMSO on day 4, *p* < 0.0001, h^2^—D3T compare to DMSO on day 5, *p* < 0.0001, h^3^—D3T compare to DMSO on day 6, *p* < 0.0001, i—Pre-D3T+D3T compare to DMSO on day 3, *p* < 0.0001, i^1^—Pre-D3T+D3T compare to DMSO on day 4, *p* < 0.0001, i^2^—Pre-D3T+D3T compare to DMSO on day 5, *p* < 0.0001, i^3^—Pre-D3T+D3T compare to DMSO on day 6, *p* < 0.0001.

To evaluate the effects of D3T on ameliorating psoriasis symptoms in vivo, an IMQ-induced psoriasis BALB/c mice model was used. Scaling, ear redness, and ear thickness were scored respectively from Day 1 to Day 6. The experimental protocol is depicted in Figure 1A, and representative photographs of mice backs are presented in Figure 1B. Psoriasis-like dermatitis was observed on Day 3 in the vehicle control group during the experiment. Compared to the vehicle control group, decreasing scores of scaling, ear redness, and ear thickness were observed in the D3T- and dexamethasone-treated groups (Figure 1C). These finding indicate that D3T could significantly alleviate IMQ-induced psoriasis in BALB/c mice. To further investigate the kinetic effects of D3T on reducing psoriasis symptoms, D3T was administered at different time points during the IMQ-induced psoriasis. The protocol is shown in Figure 2A, and representative photographs of mice backs are displayed in Figure 2B. Pre-treatment of D3T for 3 days followed by DMSO treatment for 5 days delayed onset of psoriasis and reduced ear thickness compared to the DMSO group. In the co-treatment D3T group, as well as in the pre-treatment D3T group where D3T was administered for 3 consecutive days before the D3T treatment, there was a significant reduction in skin redness, scaling, and ear thickness compared to the DMSO group. However, there was no significant differences between these two groups (Figure 2C). These data showed the pharmacodynamics of D3T in treating the psoriasis murine model.

### 2.2. Histological Examination

**Figure 3:**
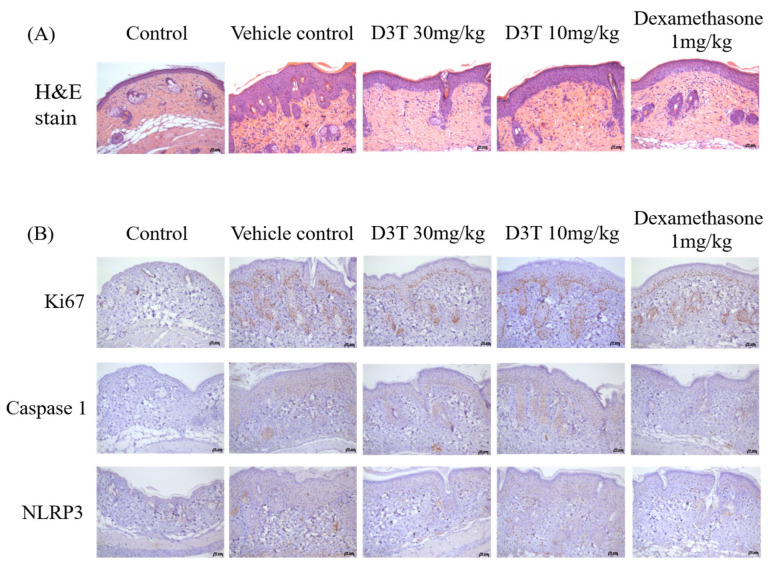
D3T decreased acanthosis, immune cell infiltration, and NLRP3 inflammasome expression in the IMQ-induced psoriasis mouse models. (**A**) The dorsal skin samples were stained with hematoxylin and eosin (magnification 200×). (**B**) Expression of ki-67, NLRP3, and caspase-1 from the dorsal skin samples were detected using IHC staining (magnification 200×).

To further investigate the effects of D3T on treating IMQ-induced psoriasis through inhibiting NLRP3 inflammasome activation, H&E staining and IHC were used to detect acanthosis and NLRP3 inflammasome expression. In the vehicle treatment group, the H&E stain showed dramatic epidermal thickness when compared to the control group. In the D3T and dexamethasone treatment group, epidermal thickness had obviously decreased when compared to the vehicle treatment group (Figure 3A). The ki-67 IHC staining revealed that keratinocyte proliferation had increased in the vehicle control group when compared to the control group. In the D3T and dexamethasone treatment group, ki-67 expression had decreased when compared with the vehicle control treatment group (Figure 3B). Furthermore, the expressions of NLRP3 inflammasome and caspase-1 were reduced in the D3T treatment group when compared to the vehicle control group (Figure 2B). These data demonstrate that D3T inhibits psoriatic symptoms by decreasing keratinocyte proliferation and NLRP3 inflammasome activation.

### 2.3. D3T Could Decrease IL-17A Expression and Th17 Cell Differentiation

**Figure 4:**
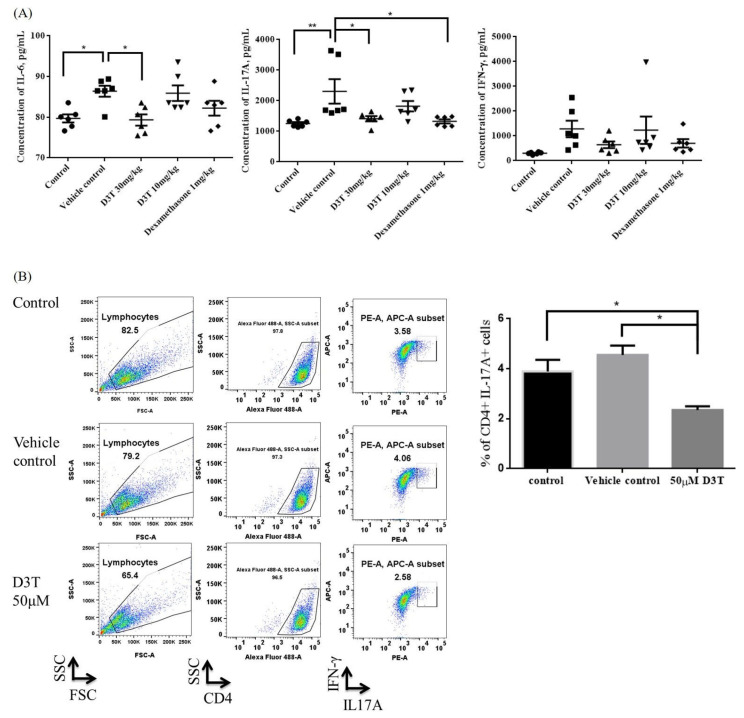
D3T downregulated the levels of IL-6 and IL-17A, and inhibited Th17 differentiation. (**A**) Concentrations of IL-6, IL-17A, and IFN-γ in the serum sample were measured by ELISA. (**B**) Naïve CD4 cells were isolated from the splenocytes of the BALB/c mice, then stimulated with monoclonal antibodies and recombinant proteins for 4 days. In the experiment groups, cells were co-cultured with 50 μM D3T. The expression of intracellular IL-17A and IFN-γ was detected using a flow cytometer (n = 3). * *p* < 0.05, ** *p* < 0.01.

The serum samples derived from mice blood were used to measure the concentrations of IL-17A, IFN-γ, and IL-6. The results show that the levels of IL-17A and IL-6 significantly decreased in the D3T treatment group when compared to the vehicle control treatment group, but the level of IFN-γ showed no difference between the groups (Figure 4A). It has been reported that inflammasome activation is correlated to Th17 cell differentiation [11], therefore, we further investigated the effects of D3T on regulating Th17 cell differentiation in vitro. The results show that after treatment with 50 μM D3T, naïve T cell differentiated to Th17 cell had been significantly reduced (Figure 4B). These data suggest that D3T could also decrease proinflammatory cytokine production and inhibit Th17 cell expression.

### 2.4. D3T Downregulating NLRP3 Inflammasome through Inhibit JNK Activation

**Figure 5:**
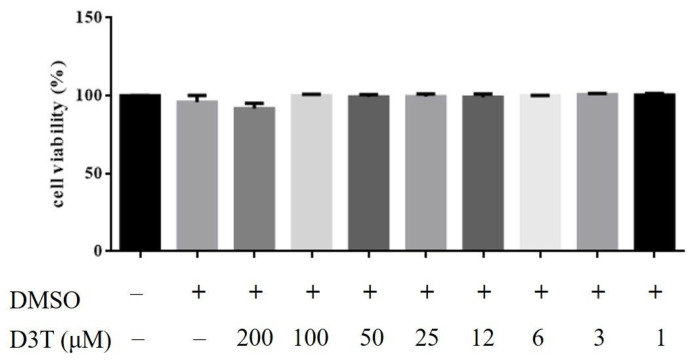
Cell viability of D3T.HaCaT cells were treated with different doses of D3T for 24 h, with cell viability measured by MTT assay.

**Figure 6:**
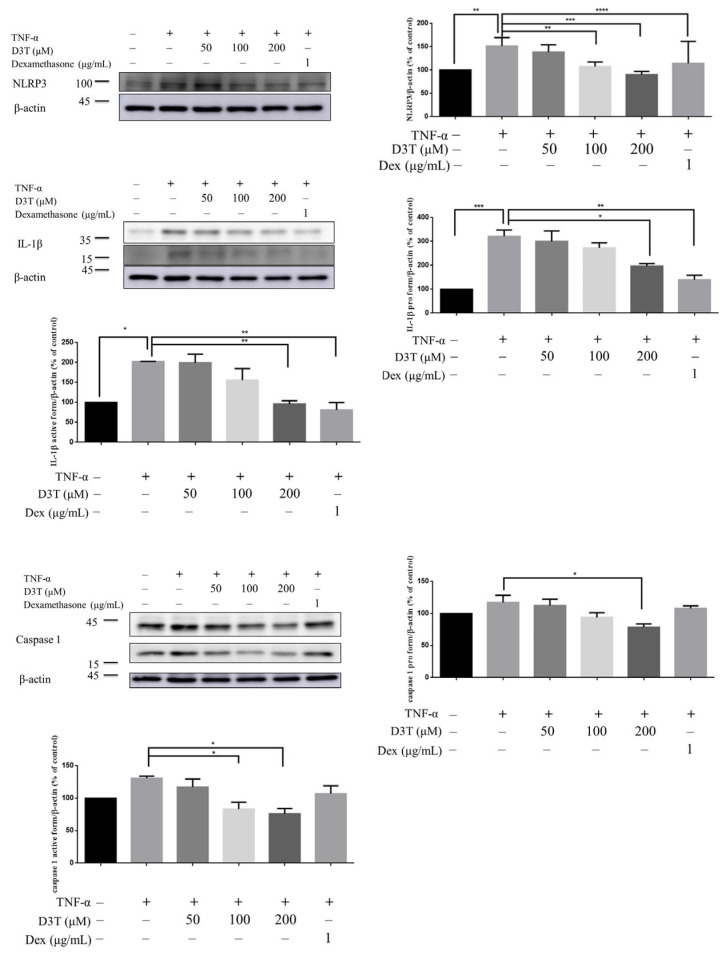
Effects of D3T on NLRP3 inflammasome activation. HaCaT Cells were co-treated with 10 ng/mL TNF-α and different doses of D3T for a period of 6 h. The expressions of NLRP3, caspase-1 (proform 45 kDa, active form 17 kDa), and IL-1β (proform 31 kDa, active form 17 kDa) were detected by Western blot. The band intensity was measured by image J. The results were expressed as the mean ± SEM. * *p* < 0.05, ** *p* < 0.01, *** *p* < 0.001, **** *p* < 0.0001. Dex: Dexamethasone.

**Figure 7:**
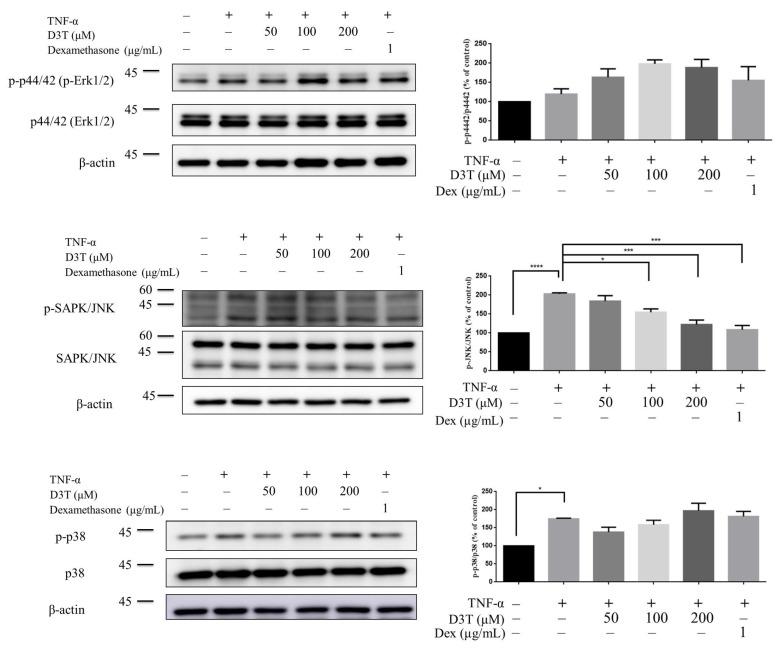
Effects of D3T on MAPK signaling pathway. HaCaT Cells were co-treated with 10 ng/mL TNF-α and different doses of D3T for 6 h. The expressions of total form and phospho form JNK, p38, and Erk1/2 were detected by Western blot. The band intensity was measured by image J (Java 1.8.0_345). The results were expressed as the mean ± SEM. * *p* < 0.05, *** *p* < 0.001, **** *p* < 0.0001. Dex: Dexamethasone.

In the mechanical study, HaCaT was used to discover the possible mechanism of D3T reducing NLRP3 inflammasome activation. First, we measured the cytotoxicity of D3T on keratinocyte, and found that a level under 200 μM of D3T would not cause cell death after 24 h of being stimulated (Figure 5). Next, we detected NLRP3 inflammasome expression after being co-cultured with TNF-α and different doses of D3T, with the expression of NLRP3 detected by Western blot. The results show that TNF-α could induce NLRP3, caspase-1, and IL-1β expression, and the expression of these proteins was downregulated in the D3T treatment group (Figure 6). Furthermore, it has been reported that the JNK signaling pathway is correlated with NLRP3 inflammasome activation [12]; therefore, we further detected MAPK protein expression through Western blot. The results show that p-JNK expression significantly decreased in the D3T treatment group (Figure 7). These data indicate that D3T could reduce proinflammatory cytokine-induced NLRP3 inflammasome activation through inhibiting the JNK pathway.

## 3. Discussion

In the past few years, the development of biologics targeting IL-17, IL-23, and TNF-α has dramatically enhanced the anti-psoriatic effect. However, the financial burden and possible adverse effects of the application of biologics has decreased patient compliance and treatment efficacy. Thus, any discovery of novel medications and new targets can provide alternative opportunities towards treating psoriasis. In this study, we showed the in vivo and pharmacokinetic evidence of D3T which could decrease ear thickness, redness, and scaling in IMQ-induced psoriasis. Furthermore, skin proliferation and NLRP3 inflammasome expression in skin samples were also downregulated in the D3T treatment group. Through an ELISA experiment, we demonstrated that D3T inhibited IL-17A and IL-6 production in serum samples. We also provided in vitro evidence that D3T could inhibit Th17 cell differentiation. In keratinocyte cell culture experiments, we demonstrated that TNF-α could induce NLRP3 inflammasome and MAPK signaling pathway activation. Additionally, after being co-cultured with D3T, we found that D3T could decrease NLRP3, caspase-1, and IL-1β expression by inhibiting JNK signaling pathway activation. For the development of new drugs, using an animal model to investigate the effects of new drugs on treating certain diseases remains necessary. It has been reported that murine models have been used to investigate psoriasis for more than 20 different types of applications, and that upon further analysis of these models, mostly surrounding gene modification, spontaneous, xenotransplantation, and inducible models, the most commonly used murine model was IMQ-induced psoriasis [13]. In this model, the experimental mice experienced the typical symptoms of psoriasis, including increased thickening of the epidermal layer and hyperkeratosis, skin inflammation, and immune cells overactivation. In the present study, we also observed similar manifestations, such as increased skin redness, scaling, acanthosis, elongation of rete-like ridges, hyperkeratosis, increased levels of IL-6 and IL-17A, and acanthosis. Therefore, this model could be used to evaluate the treatment effect of our candidate drug, D3T, on ameliorating IMQ-induced psoriasis. In this study, we demonstrated that D3T could alleviate IMQ-induced skin inflammation and its related symptoms, while also providing the necessary in vivo data regarding the treatment efficacy of D3T on psoriasis.

In the pathogenesis of psoriasis, overactivation of immune cells such as Th17 cells, dendritic cells, γδT cells, and keratinocytes are correlated with the disease onset and progression. Due to pathological immune cells overactivation, proinflammatory cytokines such as IL-17A, TNF-α, and IL-6 are secreted by the immune cells. In this process, inflammasomes play an important role in expression of proinflammatory cytokines. Inflammasomes are protein complexes composed of sensors, adaptors, and zymogens [14]. The sensor protein of inflammasomes can respond to the pathogen associate as well as dangerous associate signals such as bacteria, virus, and proinflammatory cytokines [15]. In the context of psoriasis pathogenesis, infections have been identified as potential triggers that incite immune cell overactivation, resulting in the generation of proinflammatory cytokines which stimulate keratinocyte hyperproliferation, as well as enhancing immune cells activation and infiltration into the dermis [16]. Amongst these different inflammasomes, the NLRP3 inflammasome has been reported as having an expression that was 3.5- to 4.3-fold in patients with psoriasis when compared to patients providing normal skin samples [17]. Therefore, NLRP3 has the potential to become a novel target when treating psoriasis. In the present study, we demonstrated that IMQ-induced psoriasis could induce NLRP3 and caspase-1 expression and be downregulated through D3T treatment. This suggests that D3T could be used to treat NLRP3-inflammasome-overactivation-induced diseases such as psoriasis.

In the pathogenesis of psoriasis, Th17 plays an important role in effecting immune response. Increased Th17 cells and related cytokine expression such as IL-17and IL-22 have been reported in psoriasis patients [18,19]. Therefore, targeting Th17 cell activation and its related cytokines expression are important in treating psoriasis. Previous reports showed that D3T could inhibit Th17 cells differentiation in EAE and psoriasis animal models [9,20]. Furthermore, NLRP3 inflammasome activation has been reported, which could modulate Th17 cell differentiation in patients with rheumatoid arthritis [11]. In the present study, naïve T cells differentiated to Th17 cells could be inhibited through D3T treatment, suggesting that regulation of D3T on Th17 cell differentiation may occur through inhibiting NLRP3 inflammasome activation, although the possible mechanism still requires further investigation.

To clarify the signal pathway of D3T on modulating NLRP3 inflammasome activation, mitogen-activated protein kinase (MAPK) expression was our main target of investigation. The MAPK pathway, including JNK, ERK, and p38, is important for the production of proinflammatory cytokines such as IL-6 and IL-8. Moreover, MAPK pathway overactivation has been reported in the skin lesions of psoriasis patients [21]. TNF-α and IL-17A both play a major role in inducing keratinocyte MAPK pathway activation in patients with psoriasis [22,23]. Furthermore, MAPK pathway activation is correlated with keratinocyte AIM2 inflammasome activation [24]. In the present study, we demonstrated that D3T could inhibit TNF-α-induced p-JNK expression, suggesting that the inhibition of NLRP3 inflammasome activation by D3T may downregulate JNK pathway activation.

Although we demonstrated the treatment effect of D3T on reducing NLRP3 inflammasome activation in IMQ-induced psoriasis and the keratinocyte cell line, there are still some limitations to this study. First, in this study D3T treatment was performed through an intraperitoneal injection. Based on the currently available studies, both topical [20] and systemic administrations are associated with positive results. In a study by Brown et al., reduced activity, lethargy, rough coat, and piloerection were observed in rodent models in groups that received oral doses of 20–60 mg/kg/day for a duration of 14 days. The severity of these symptoms was found to be dosage dependent [25]. From the literature review, we acknowledge the limitation of not having yet identified the optimal route and dosage for ensuring safety and effectiveness. Thus, further determination of the optimal route and dosage should be investigated in the future. Second, in this study, we demonstrated the effects of D3T on psoriasis in animal models and the HaCaT cell line. Clinical trials and primary skin samples should be used to further investigate the clinical effect of D3T on psoriasis in the future. 

## 4. Materials and Methods

### 4.1. Mouse Model for Imiquimod (IMQ)-Induced Psoriasis-like Skin Inflammation

Female mice BALB/c (8 weeks) were obtained from the National Laboratory Animal Center (Taipei, Taiwan). The following animal protocol has been approved by the Institutional Animal Care and Use Committee of Taichung Veterans General Hospital (La-1111908; La-1121951). The BALB/c mice were housed on well-ventilated racks in a temperature- and humidity-controlled room under a regular light/dark cycle (light:dark = 12 h:12 h), and all had free access to rodent chow and tap water. Mice were randomly divided into five groups of six mice each. The groups included a wild type control, vehicle control (dimethyl sulfoxide, DMSO, Sigma-Aldrich, St. Louis, MI, USA), dexamethasone (1 mg/kg/day), D3T (Abcam, Fremont, CA, USA; 10 mg/kg/day), and D3T (Abcam, USA; 30 mg/kg/day). DMSO, dexamethasone, and D3T were administrated through intraperitoneal injection (i.p.), while 62.5 mg IMQ topical cream (Aldara^®^, Somerset, NJ, USA) was applied to the shaved dorsal areas and ears for a period of five consecutive days. Psoriasis severities, including redness, scaling, ear thickness, and body weight were all evaluated daily prior to IMQ application. Scores were interpreted as follows: 0 = none, 1 = mild, 2 = moderate, 3 = severe, and 4 = very severe. The thickness of each mice ear was measured using a digital thickness gauge. 

### 4.2. Histological and Immunohistochemical Stain

The dorsal skin of each mouse was cut and fixed in a formalin solution, embedded in paraffin, and cut into a 3 μm section before being stained with hematoxylin and eosin (H&E). For immunohistochemical staining, after paraffin removal, antigen retrieval, and blocking, the 3 μm sample sections were incubated for forty minutes at room temperature with ki-67, NLRP3, and caspase-1 primary antibodies (Abcam, Fremont, CA, USA, AdipoGen, San Diego, CA, USA). After washing, the sections were incubated with an antibody enhancer (GBI lab; Rockville, MD, USA) for ten minutes at room temperature. After another washing, the sections were then incubated with horseradish peroxidase (HRP)-conjugated antibodies (GBI lab) for ten minutes at room temperature. After a third washing, the sections were developed with DAB (Thermo Fisher Scientific, Waltham, MA, USA) for two minutes at room temperature and counterstained with hematoxylin. A sample section was visualized under a digital camera (AxioCam HRc, Oberkochen, Germany) which had been mounted on a phase contrast microscope (AXIOVERT 200M, Zeiss, Oberkochen, Germany) using AxioVision Rel. 4.8 software (Zeiss, Oberkochen, Germany).

### 4.3. Enzyme-Linked Immunosorbent Assay (ELISA)

The concentrations of IL-17A, IL-6, and IFN-γ from the serum samples of the experimental mice were measured using the sandwich ELISA method, following the manufacturer’s protocol (BioLegend, San Diego, CA, USA). Absorbance at 450 nm was measured using a multiplate reader (PerkinElmer, Waltham, MA, USA).

### 4.4. Isolation of CD4+T Cells and Th17 Polarization

CD4+T cells were purified from single-cell suspensions of BALB/c mouse spleens using the EasySep™ Mouse CD4+T Cell Isolation Kit (STEMCELL, Cambridge, MA, USA). After isolation, the cells were seeded on 96-well plates at a concentration of 3 × 10^5^ cells/well with an IMDM medium (Gibco, Waltham, MA, USA), supplemented with 5% heat inactivated fetal bovine serum (Cytiva, Marlborough, MA, USA), NCAA, 2-mercaptoethanol, sodium pyruvate, and 100 U/mL penicillin, as well as 100 μg/mL streptomycin (Gibco, Waltham, MA, USA) at 37 °C using a 5% CO_2_ incubator. The isolated CD4+T cells were activated with a CellXVivo Mouse Th17 Cell Differentiation Kit (R&D Systems, Minneapolis, MN, USA) following the manufacture’s protocol. To evaluate the effects of D3T on Th17 cell differentiation, D3T was added to the Th17 polarizing medium and incubated for four days. 

### 4.5. Flow Cytometry Analysis

Samples from the Th17 cell differentiation experiments were stimulated with ionomycin (500 ng/mL; Sigma-Aldrich, St. Louis, MI, USA), phorbol 12-myristate 13-acetate (50 ng/mL; Sigma-Aldrich, USA), and monensin (BD, Franklin Lakes, NJ, USA) for a period of five hours. Anti-mouse CD4 (BioLegend, San Diego, CA, USA) was used to stain the cell surface marker before it was incubated with samples for fifteen minutes at 4 °C. After washing, samples were fixed and permeabilized using a Cytofix/Cytoperm kit (BD, Franklin Lakes, NJ, USA). After washing again, the intracellular cytokines were stained with anti-IFN-γ and IL-17A (BioLegend, San Diego, CA, USA) for thirty minutes at 4 °C. The samples were then acquired on a flow cytometer (FACSCanto II; BD, Franklin Lakes, NJ, USA), with the data analyzed using FlowJo software v10.8.1 (Tree Star, Ashland, OR, USA).

### 4.6. Cell Culture

A human keratinocyte HaCaT cell line was purchased from Elabscience (Elabscience Biotechnology Inc, Houston, TX, USA). The HaCaT cells were cultured in Dulbecco’s modified Eagle’s medium (DMEM; Gibco, Waltham, MA, USA), supplemented with 10% heat-inactivated fetal bovine serum (Cytiva, Marlborough, MA, USA) and 1% penicillin-streptomycin (Gibco, Waltham, MA, USA), and ultimately maintained in a humidified incubator containing 5% CO_2_ at 37 °C. 

### 4.7. Immunoblotting

The HaCaT cells were co-stimulated with TNF-α (10 ng/mL; PeproTech; USA) and different doses of D3T for either 6 or 24 h at 37 °C in an incubator. After incubation, total protein was extracted in a RIPA lysis buffer (Bio Basic, Markham, ON, Canada) with protease and phosphatase inhibitors (Bionovas, Toronto, Ontario, Canada; TargetMol, Wellesley Hills, MA, USA). Total protein was quantified by a Coomassie (Bradford) Protein Assay Kit (Thermo Fisher Scientific, Waltham, MA, USA). Protein samples were separated through a 10% SDS-PAGE and transferred to a PVDF membrane. Primary antibodies against p-ERK, ERK, p-JNK, JNK, p-p38, p38, NLRP3, caspase-1, IL-1β, and β-actin (Cell signaling, Danvers, MA, USA; GeneTex, Hsinchu City, Taiwan) were incubated with a PVDF membrane overnight at 4 °C. The following day, TTBS was used to wash the membrane three times prior to incubation with secondary antibodies, which were then conjugated with horseradish peroxidase for one hour at room temperature. After using TTBS to wash the membrane three times, its surface was covered with enhanced chemiluminescence (ECL; Merck Millipore, Danvers, MA, USA). The signal was detected by the Amersham Imager 680 (Cytiva, Marlborough, MA, USA).

### 4.8. Statistical Analysis

Phenotype data taken from the animal experiments, including ear thickness, ear redness, and scaling, were analyzed by two-way ANOVA followed by a Tukey multiple comparison test. All other data were analyzed through one-way ANOVA followed by a Tukey multiple comparison test. A *p* < 0.05 was considered statistically significant, with data being analyzed with the GraphPad Prism 6.0 (GraphPad Software, Boston, MA, USA).

## 5. Conclusions

In this study, we demonstrated the effects of D3T on inhibiting NLRP3 inflammasome activation in the treatment of psoriasis through in vivo and in vitro experiments. In the in vivo experiments, we demonstrated that D3T could ameliorate IMQ-induced psoriasis and reduce NLRP3 inflammasome activation, and in the in vitro experiments we demonstrated that D3T could inhibit Th17 cell differentiation and decrease TNF-α-induced NLRP3 inflammasome expression and JNK pathway activation. Through this study, we have provided evidence that D3T has a novel molecular targeting ability for treating psoriasis, which can be further developed for the treatment of inflammasome overactivation diseases.

## Data Availability

Data sharing is not applicable to this article.

## References

[1] Michalek I., Loring B., John S. (2017). A systematic review of worldwide epidemiology of psoriasis. J. Eur. Acad. Dermatol. Venereol..

[2] Pirowska M., Obtulowicz A., Lipko-Godlewska S., Gozdzialska A., Podolec K., Wojas-Pelc A. (2018). The level of proinflammatory cytokines: Interleukins 12, 23, 17 and tumor necrosis factor alpha in patients with metabolic syndrome accompanying severe psoriasis and psoriatic arthritis. Postepy Dermatol. Alergol..

[3] Swanson K.V., Deng M., Ting J.P. (2019). The NLRP3 inflammasome: Molecular activation and regulation to therapeutics. Nat. Rev. Immunol..

[4] Kelley N., Jeltema D., Duan Y., He Y. (2019). The NLRP3 Inflammasome: An Overview of Mechanisms of Activation and Regulation. Int. J. Mol. Sci..

[5] Tang L., Zhou F. (2020). Inflammasomes in Common Immune-Related Skin Diseases. Front. Immunol..

[6] Hooftman A., Angiari S., Hester S., Corcoran S.E., Runtsch M.C., Ling C., Ruzek M.C., Slivka P.F., McGettrick A.F., Banahan K. (2020). The Immunomodulatory Metabolite Itaconate Modifies NLRP3 and Inhibits Inflammasome Activation. Cell Metab..

[7] Brown D.A., Betharia S., Yen J.-H., Tran Q., Mistry H., Smith K. (2014). Synthesis and structure–activity relationships study of dithiolethiones as inducers of glutathione in the SH-SY5Y neuroblastoma cell line. Bioorganic Med. Chem. Lett..

[8] Zhu H., Jia Z., Zhang L., Yamamoto M., Misra H.P., Trush M.A., Li Y. (2008). Antioxidants and Phase 2 Enzymes in Macrophages: Regulation by Nrf2 Signaling and Protection against Oxidative and Electrophilic Stress. Exp. Biol. Med..

[9] Kuo P.-C., Brown D.A., Scofield B.A., Yu I.-C., Chang F.-L., Wang P.-Y., Yen J.-H. (2016). 3H-1,2-dithiole-3-thione as a novel therapeutic agent for the treatment of experimental autoimmune encephalomyelitis. Brain, Behav. Immun..

[10] Hu R., Wang M.Q., Ni S.H., Wang M., Liu L.Y., You H.Y., Wu X.H., Wang Y.J., Lu L., Wei L.B. (2020). Salidroside ameliorates endothelial inflammation and oxidative stress by regulating the AMPK/NF-κB/NLRP3 signaling pathway in AGEs-induced HUVECs. Eur. J. Pharmacol..

[11] Zhao C., Gu Y., Zeng X., Wang J. (2018). NLRP3 inflammasome regulates Th17 differentiation in rheumatoid arthritis. Clin. Immunol..

[12] Chu X., Wang C., Wu Z., Fan L., Tao C., Lin J., Chen S., Lin Y., Ge Y. (2021). JNK/c-Jun-driven NLRP3 inflammasome activation in microglia contributed to retinal ganglion cells degeneration induced by indirect traumatic optic neuropathy. Exp. Eye Res..

[13] Chuang S.-Y., Lin C.-H., Sung C.T., Fang J.-Y. (2018). Murine models of psoriasis and their usefulness for drug discovery. Expert Opin. Drug Discov..

[14] Sharma D., Kanneganti T.-D. (2016). The cell biology of inflammasomes: Mechanisms of inflammasome activation and regulation. J. Cell Biol..

[15] Franchi L., Eigenbrod T., Muñoz-Planillo R., Nuñez G. (2009). The inflammasome: A caspase-1-activation platform that regulates immune responses and disease pathogenesis. Nat. Immunol..

[16] Zhou S., Yao Z. (2022). Roles of Infection in Psoriasis. Int. J. Mol. Sci..

[17] Su F., Xia Y., Huang M., Zhang L., Chen L. (2018). Expression of NLPR3 in Psoriasis Is Associated with Enhancement of Interleukin-1β and Caspase-1. Med. Sci. Monit. Int. Med. J. Exp. Clin. Res..

[18] Fitch E., Harper E., Skorcheva I., Kurtz S.E., Blauvelt A. (2007). Pathophysiology of psoriasis: Recent advances on IL-23 and Th17 cytokines. Curr. Rheumatol. Rep..

[19] Schon M.P., Erpenbeck L. (2018). The Interleukin-23/Interleukin-17 Axis Links Adaptive and Innate Immunity in Psoriasis. Front. Immunol..

[20] Liu C.T., Yen J.J., Brown D.A., Song Y.C., Chu M.Y., Hung Y.H., Tang Y.H., Wu P.Y., Yen H.R. (2023). Targeting Nrf2 with 3H-1,2-dithiole-3-thione to moderate OXPHOS-driven oxidative stress attenuates IL-17A-induced psoriasis. Biomed. Pharmacother..

[21] Haase I., Hobbs R.M., Romero M.R., Broad S., Watt F.M. (2001). A role for mitogen-activated protein kinase activation by integrins in the pathogenesis of psoriasis. J. Clin. Investig..

[22] Furue M., Furue K., Tsuji G., Nakahara T. (2020). Interleukin-17A and Keratinocytes in Psoriasis. Int. J. Mol. Sci..

[23] Szentkereszty-Kovács Z., Gáspár K., Szegedi A., Kemény L., Kovács D., Törőcsik D. (2021). Alcohol in Psoriasis—From Bench to Bedside. Int. J. Mol. Sci..

[24] Cao T., Yuan X., Fang H., Chen J., Xue K., Li Z., Dang E., Wang G., Shao S. (2023). Neutrophil extracellular traps promote keratinocyte inflammation via AIM2 inflammasome and AIM2-XIAP in psoriasis. Exp. Dermatol..

[25] Brown A.P., Morrissey R.L., Tolhurst T.A., Crowell J.A., Levine B.S. (2000). Oral Toxicity of 1,2-Dithiole-3-Thione, a Potential Cancer Chemopreventive Agent, in the Rat. Int. J. Toxicol..

